# Use of appendicostomy in the management of necrotizing enterocolitis: a case report

**DOI:** 10.1097/RC9.0000000000001042

**Published:** 2026-09-21

**Authors:** Sidikov Odilbek, Mirzakarimov Bahrom, Ajimamatov Halilullo, Toshboev Sherzod

**Affiliations:** a Department of Pediatric Surgery, Andijan State Medical Institute, Andijan, Uzbekistan; b Department of Anaesthesiology, Reanimation and Emergency Medicine, Andijan State Medical Institute, Andijan, Uzbekistan

**Keywords:** appendicostomy, bowel decompression, case report, necrotizing enterocolitis, neonatal surgery, pneumoperitoneum

## Abstract

**Introduction and importance:**

Necrotizing enterocolitis (NEC) complicated by pneumoperitoneum usually mandates emergency laparotomy. When no intestinal perforation or transmural necrosis is found, the optimal surgical strategy remains undefined. Conventional options – planned second-look laparotomy, primary peritoneal drainage, or proximal enterostomy – each carries significant burdens, and high-output stomas are particularly hazardous in premature neonates. We describe a decompressive tube appendicostomy used as a pressure-relief conduit in this uncommon scenario.

**Presentation of the case:**

An 8-day-old premature male infant (34 weeks; 2850 g) underwent emergency laparotomy for NEC with pneumoperitoneum (Bell stage IIIB); preoperative heart rate was 135/min, oxygen saturation was 95%, and blood pressure was 56/34 mmHg. Findings included diffuse small-bowel pneumatosis with patchy subserosal hemorrhagic discoloration, without transmural necrosis or identifiable perforation; the colon was macroscopically normal. A decompressive tube appendicostomy was fashioned through a right transrectal incision: a 10 Fr catheter was passed through the appendiceal lumen and advanced across the ileocecal valve approximately 6 cm into the terminal ileum, with intraluminal position confirmed by manual palpation, providing direct small-bowel decompression, while peri-catheter effluent decompressed the cecum. No proximal enterostomy or bowel resection was performed.

**Clinical discussion:**

The catheter was removed on postoperative day (POD) 5, and enteral feeding was reintroduced on POD 7. The appendicostomy closed spontaneously by week 8 without reversal surgery. No dehydration, electrolyte disturbance, or stomal complication occurred on routine postoperative monitoring. Because direct ileal decompression was achieved, the construct functioned as a low-resistance vent rather than a diverting stoma.

**Conclusion:**

A decompressive tube appendicostomy may serve as a pressure-relief conduit in carefully selected neonates with NEC and pneumoperitoneum, when no transmural perforation is found. Its independent contribution to recovery cannot be established from a single, uncontrolled observation; a prospective study is required.

## Introduction

Necrotizing enterocolitis (NEC) is a life-threatening gastrointestinal emergency of premature, low-birth-weight infants, characterized by intestinal inflammation, ischemia, and progressive mucosal injury^[^1,2^]^. Up to one-third of affected neonates require operative treatment, and mortality in this subgroup remains high^[^3,4^]^. In NEC complicated by pneumoperitoneum, perforation is generally assumed; occasionally, however, laparotomy reveals diffuse pneumatosis and hemorrhagic changes without an identifiable transmural defect^[^5,6^]^. Conventional options then include primary abdominal closure with planned second-look laparotomy, primary peritoneal drainage (PPD), or proximal enterostomy^[^7–9^]^, each with drawbacks: repeat intervention, repeated anesthesia in an unstable neonate, or high-output losses requiring reversal surgery^[^7,8^]^. Appendicostomy is established for antegrade colonic irrigation but has not, to our knowledge, been described as a decompressive pressure-relief conduit in neonatal NEC. We report such use in a premature neonate with NEC, pneumoperitoneum, and no identifiable perforation at laparotomy. This case is presented in line with the SCARE 2025 criteria^[^10^]^.

HIGHLIGHTS

- Necrotizing enterocolitis with pneumoperitoneum may occur without an identifiable bowel perforation.
- A tube appendicostomy can act as a pressure-relief vent, not as a diverting stoma.
- A transvalvular catheter provided direct ileal decompression, sparing a proximal stoma.
- Spontaneous closure of the appendicostomy at 8 weeks required no reversal surgery.
- A single hypothesis-generating case; a prospective study is needed before adoption.

## Presentation of case

### Patient information, findings and severity

An 8-day-old premature male (gestational age 34 weeks; birth weight 2850 g) was referred for suspected NEC. Formula feeding had begun at birth owing to maternal lactation difficulties. On day 5 of life, abdominal distension developed; a nasogastric tube was placed, and broad-spectrum antibiotics were commenced. By day 7, there were blood-streaked stools and thrombocytopenia, and abdominal ultrasound showed intestinal pneumatosis with reduced motility. On day 8, distension worsened acutely with a hemorrhagic gastric aspirate, and upright radiography confirmed free subdiaphragmatic air (Fig. 1). Preoperatively, the infant had a heart rate of 135 beats per minute, oxygen saturation of 95%, blood pressure of 56/34 mmHg, and a temperature of 37.2°C. Investigations showed thrombocytopenia (platelet count 80 × 10^9^/L), C-reactive protein of 16 mg/L, white-cell count of 12 × 10^9^/L and blood-gas pH of 7.35. Bell stage IIIB was assigned on the basis of pneumoperitoneum together with clinical deterioration. Empirical antibiotics comprised amikacin 61 mg once daily (gram-negative enteric coverage) and metronidazole 52 mg twice daily (anaerobic coverage), continued for a total of 10 days. Emergency laparotomy was undertaken under general anesthesia.

**Figure 1.**
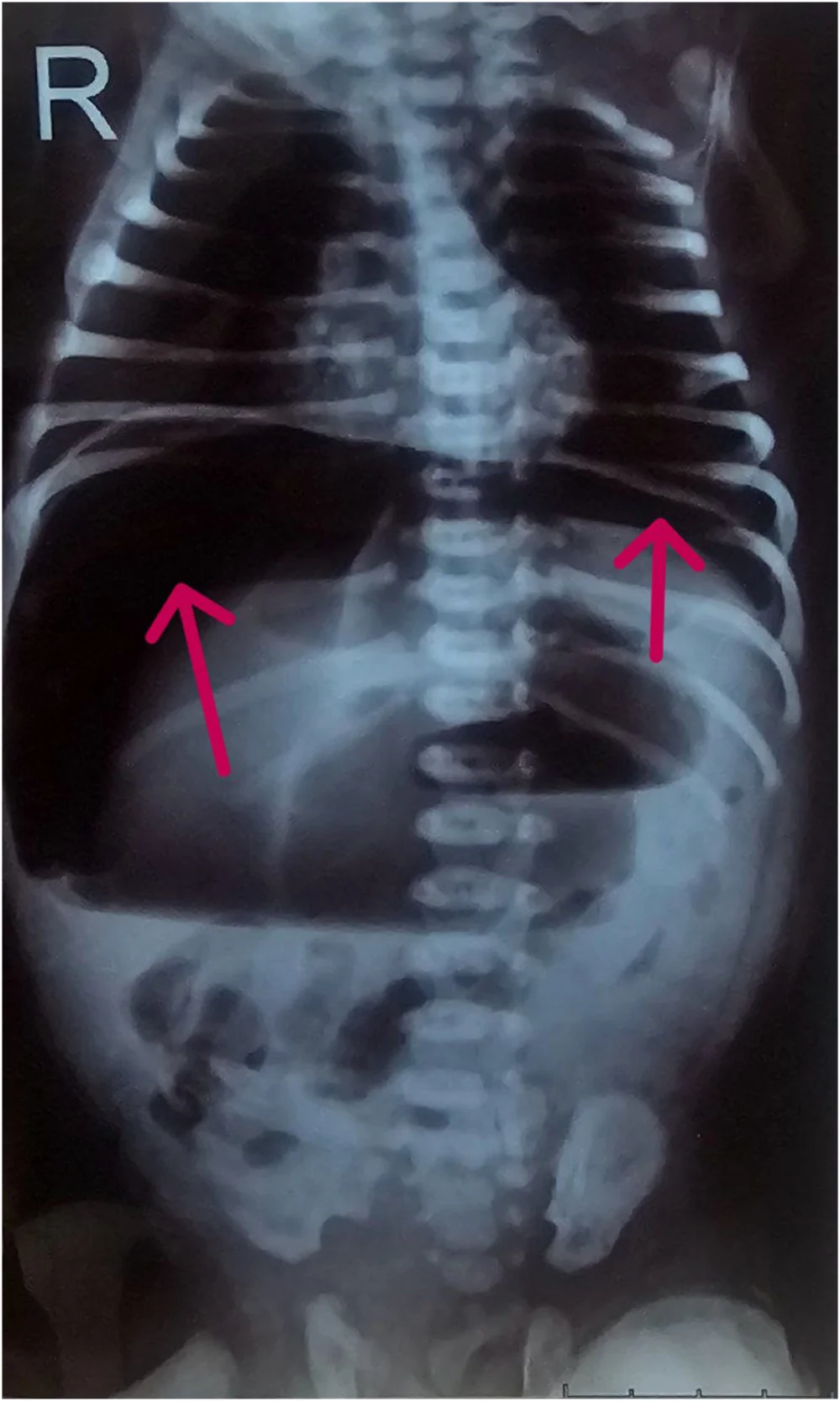
Pre-operative upright abdominal radiograph demonstrating free subdiaphragmatic air (pneumoperitoneum) with distended bowel loops (arrows).

### Intraoperative findings and technique

The procedure was performed by a senior attending pediatric surgeon holding the highest professional qualification category, with 10 years of surgical experience, at a regional pediatric multidisciplinary medical center that also functions as a scientific-research institution, with established experience in neonatal surgical emergencies. Systematic inspection from the ligament of Treitz to the rectum revealed diffuse small-bowel pneumatosis with patchy subserosal hemorrhagic discoloration (Fig. 2A); the bowel lumen was not opened. No transmural necrosis or discrete perforation was identified, and the colon was macroscopically normal. The pneumoperitoneum was attributed to rupture of gas from subserosal pneumatosis or to a spontaneously sealed microperforation^[^5,6^]^. After intraoperative multidisciplinary discussion, a conservative decompressive approach was selected in place of resection or diverting enterostomy. Through a right transrectal incision, the appendix – confirmed macroscopically normal with the mesoappendix preserved – was intubated through its lumen with a 10-French Nelaton catheter; unlike a Malone/ACE appendicostomy, no submucosal anti-reflux tunnel was created (a simple non-tunneled tube appendicostomy for decompression) (Fig. 2B). The catheter was advanced across the immature, variably competent ileocecal valve, with its tip positioned approximately 6 cm within the terminal ileum, achieving direct intraluminal decompression of the diseased small bowel; effluent draining around the catheter decompressed the cecum. Correct intraluminal position of the catheter tip within the terminal ileum was confirmed intraoperatively by manual palpation through the bowel wall; no radiocontrast or fluoroscopic confirmation was performed. Intraluminal gas and inflammatory effluent were evacuated immediately. The appendix was fixed to the abdominal wall with 4/0 polyglactin, and the catheter was secured to the skin with 5/0 polydioxanone (Fig. 3A). A Penrose drain was placed, and the abdomen was closed in layers. No bowel resection or proximal enterostomy was performed.

**Figure 2.**
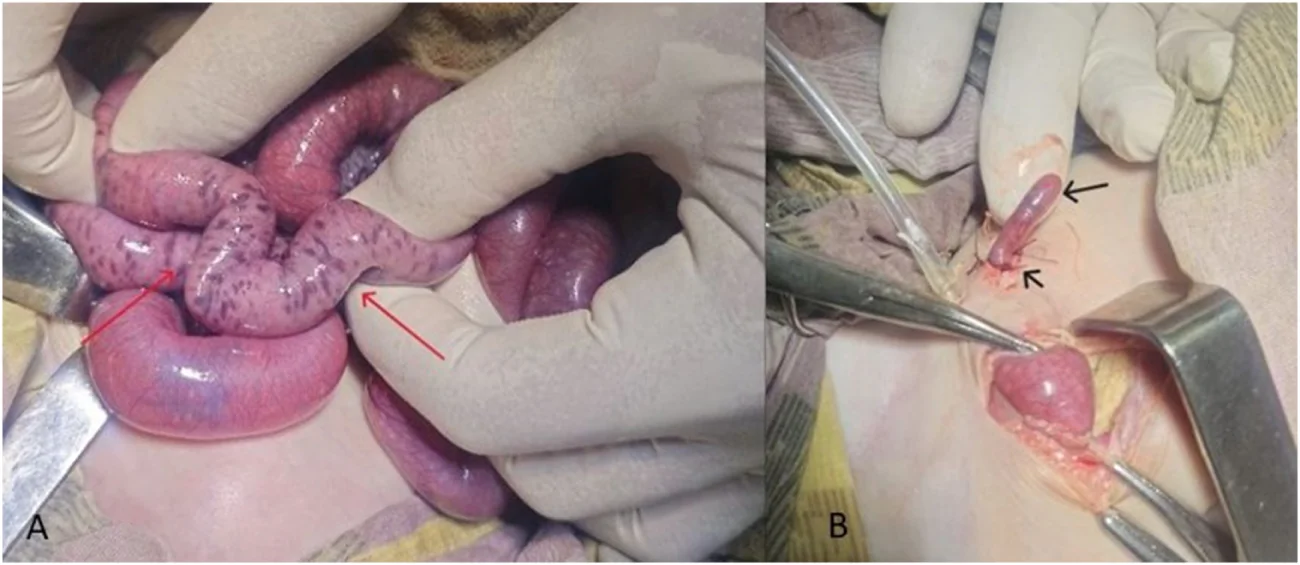
Intraoperative findings. (A) Diffuse small-bowel pneumatosis with patchy subserosal hemorrhagic discoloration; no transmural necrosis or perforation was identified (arrows). (B) Construction of the decompressive tube appendicostomy: the appendix, exteriorized through a right transrectal incision, was intubated with a 10-French catheter (arrows); the catheter was subsequently advanced across the ileocecal valve into the terminal ileum.

**Figure 3.**
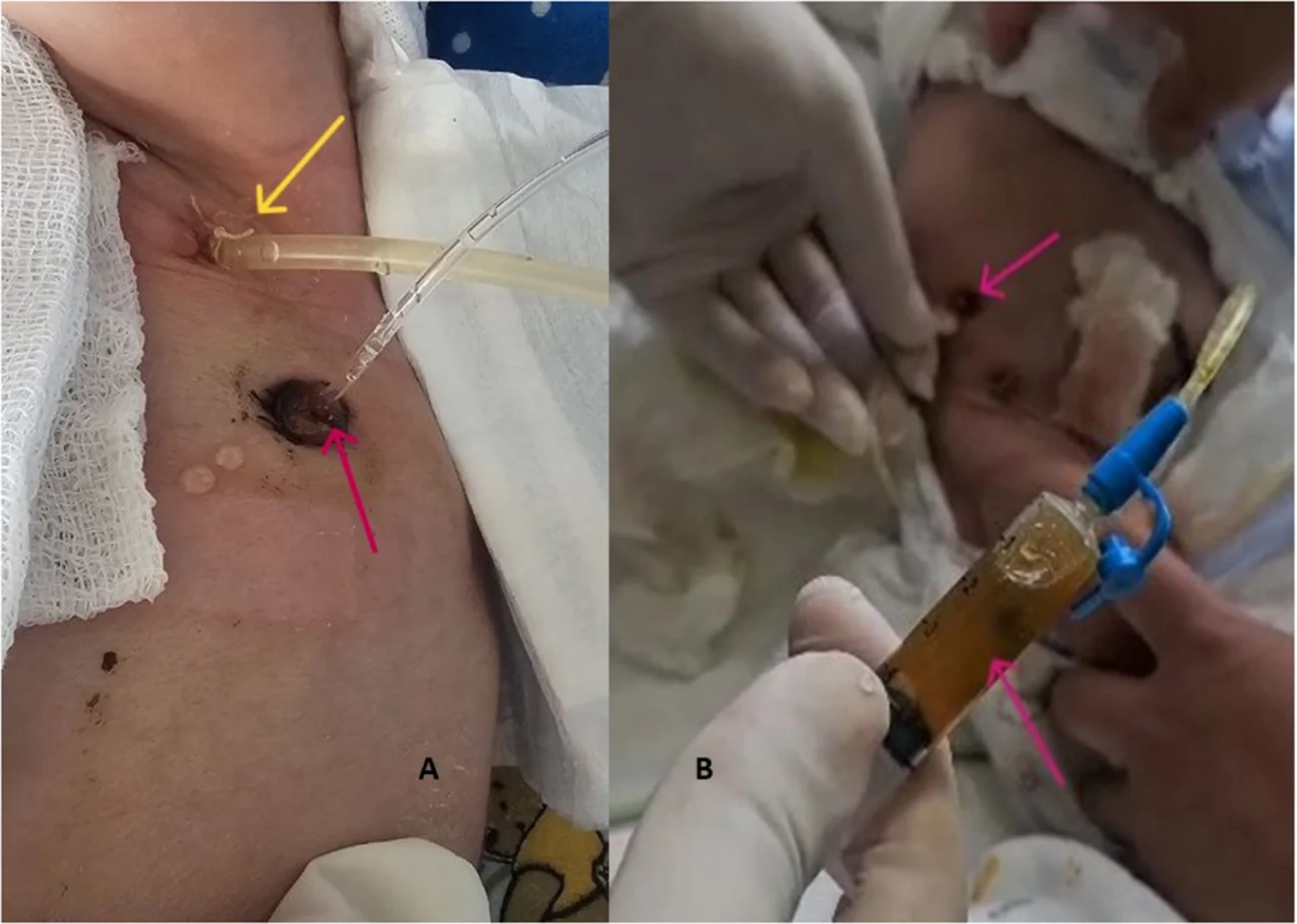
Postoperative decompression through the appendicostomy. (A) The catheter secured *in situ* to the anterior abdominal wall (arrows). (B) Syringe aspiration of bile-stained intraluminal effluent and gas (every 3 hours; approximately 5–7 mL per pass), with the bile-stained effluent consistent with the catheter tip lying within the terminal ileum.

### Postoperative course, outcomes, and follow-up

Parenteral nutrition was provided for 4 days. Abdominal distension began to subside from postoperative day (POD) 2. The appendicostomy was vented by gravity drainage with gentle syringe aspiration every 3 hours, yielding approximately 5–7 mL of effluent and gas per pass (Fig. 3B), and was irrigated with 5–10 mL of normal saline twice daily. The catheter was removed on POD 5. Graded enteral reintroduction followed, with breast-milk feeding from POD 7, which was tolerated; the patient was discharged on POD 10. Complications were defined a priori (Clavien–Dindo classification and standard stomal criteria); none occurred – no appendiceal perforation, catheter-related complication, stool impaction, excess granulation tissue, or peristomal inflammation; and no clinical evidence of dehydration or electrolyte disturbance on routine postoperative monitoring. The 30-day course was uneventful, with no readmission or reoperation. Appendicostomy output diminished by days 10–12; the stoma contracted from approximately day 40 and closed spontaneously by week 8, requiring no reversal surgery (Supplemental Digital Content Figure S1, available at: https://links.lww.com/IJSCR/A165). The clinical timeline is summarized in Table 1.

**Table 1:** Clinical timeline.

Day/Timepoint	Events
Day 5 of life	Abdominal distension; nasogastric tube placed; broad-spectrum antibiotics commenced
Day 7	Blood-streaked stools; thrombocytopenia; ultrasound—pneumatosis and hypomotility
Day 8 (surgery)	Pneumoperitoneum on radiograph; emergency laparotomy; non-tunneled tube appendicostomy (catheter advanced across ileocecal valve into terminal ileum); Penrose drain
POD 2	Abdominal distension begins to subside
POD 1–4	IV antibiotics; appendicostomy decompression; parenteral nutrition
POD 5	Decompression catheter removed
POD 7	Graded enteral reintroduction; breast-milk feeding
POD 10	Discharged home
Day ~40	Spontaneous appendicostomy contraction
Day ~60 (week 8)	Complete spontaneous closure; no further surgery

POD, postoperative day.

## Discussion

This case illustrates an uncommon intraoperative scenario: NEC with pneumoperitoneum but no identifiable transmural defect, most likely resulting from rupture of subserosal pneumatosis gas collections or spontaneous sealing of a microperforation before laparotomy^[^5,6^]^. The operative challenge lay not in resection but in achieving safe decompression while preserving intestinal length.

An appendicostomy ordinarily provides cecal – not small-bowel – access, and the pathology here was confined to the small intestine while the colon was uninvolved. In this case, however, the catheter was not left in the cecum; its tip lay approximately 6 cm within the terminal ileum, so decompression of the diseased small bowel was direct (transvalvular) rather than merely retrograde, and the construct functioned as a low-resistance intraluminal vent rather than a diverting stoma. Any additional retrograde pressure equilibration across the immature valve is a secondary, unproven consideration; no intraluminal pressures were measured.

Conventional alternatives were considered. The absence of necrosis and perforation removed the indication for resection. PPD risked leaving the distended, gas-filled bowel undrained and is generally used as a temporizing bridge^[^7,11^]^; planned second-look laparotomy would have exposed an unstable neonate to repeated anesthesia^[^8,9^]^; and a proximal jejunostomy or ileostomy would have caused high-output losses, electrolyte disturbance, and dehydration, mandating reversal and prolonging hospitalization, while the diffuse small-bowel involvement made any single enterostomy site arbitrary^[^8,12^]^. The clip-and-drop technique is reserved for borderline viability and itself requires a re-look and an anastomosis^[^13^]^. Against this background, a non-tunneled decompressive appendicostomy addressed the actual problem – intraluminal distension – directly, preserving bowel length, avoiding high-output metabolic losses, and offering spontaneous closure without reversal. The damage-control and bowel-preservation literature^[^13,14^]^ provides conceptual context only and does not directly validate appendicostomy in neonatal NEC.

Both ruptured subserosal pneumatosis and a spontaneously sealed microperforation were considered potential sources of free air, and primary peritonitis was also entertained. Peritoneal fluid was not sent for Gram stain or culture, and no dedicated primary-peritonitis protocol was followed; we acknowledge this as a limitation. The diagnosis of NEC was nonetheless well supported by the combined clinical, laboratory, imaging, and intraoperative findings.

The favorable outcome cannot be attributed to the appendicostomy in isolation; the laparotomy itself, drainage, antibiotics, bowel rest, parenteral nutrition, and supportive neonatal care all contributed, and spontaneous recovery cannot be excluded. This report is therefore positioned strictly as a descriptive, hypothesis-generating observation. Beyond the present scenario, appendicostomy might merit cautious evaluation as a palliative decompressive measure in NEC totalis where curative resection is not feasible^[^15^]^, or as a component of damage-control laparotomy in critically unstable neonates^[^14^]^; both require prospective study before adoption. Strengths include a temporary, bowel-preserving technique that closed spontaneously and its cross-specialty relevance; limitations include the single-case design, the absence of intraluminal pressure and peritoneal microbiological data, dependence on adequate appendiceal caliber (which may preclude catheterization), and the inability to establish outcome causation. Catheter position was verified intraoperatively by manual palpation rather than radiocontrast or fluoroscopic imaging, which is a further limitation; objective radiological confirmation is recommended in future applications of this technique. The technique should not be generally recommended on the basis of this report.

### Learning points

1. NEC with pneumoperitoneum can occur without an identifiable transmural perforation; a laparotomy may reveal diffuse pneumatosis without resectable necrosis.

2. A non-tunneled decompressive tube appendicostomy, with the catheter advanced across the ileocecal valve into the ileum, can provide direct small-bowel decompression while avoiding a high-output proximal stoma and may close spontaneously without reversal surgery.

3. As a single, uncontrolled observation, the technique is hypothesis-generating only; it should be reserved for carefully selected cases and evaluated prospectively before any broader adoption.

## Conclusion

In a carefully selected premature neonate with NEC and pneumoperitoneum in whom no transmural perforation was identified at laparotomy, a decompressive tube appendicostomy – with the catheter advanced transvalvularly into the terminal ileum – provided direct small-bowel decompression, avoided a high-output proximal stoma, and closed spontaneously without reversal surgery. Its independent contribution to recovery cannot be established from a single uncontrolled observation, and spontaneous recovery cannot be excluded. Prospective comparative studies with standardized outcome measures are required to define the role of this technique in neonatal NEC.

## Data Availability

Not applicable.

## Reporting guideline

Prepared in accordance with the SCARE 2025 guideline: Kerwan A, Al-Jabir A, Mathew G, Sohrabi C, Rashid R, Franchi T, Nicola M, Agha M, Agha RA; SCARE Group. Revised Surgical CAse REport (SCARE) guideline: an update for the age of Artificial Intelligence. Premier J Sci. 2025;10:100079. doi:10.70389/PJS.100079

## References

[1] Alsaied A Islam N Thalib L. Global incidence of necrotizing enterocolitis: a systematic review and meta-analysis. BMC Pediatr 2020;20:344. 10.1186/s12887-020-02231-5 PMC7359006 32660457

[2] Patel RM Ferguson J McElroy SJ. Defining necrotizing enterocolitis: current difficulties and future opportunities. Pediatr Res 2020;88:10–15. 10.1038/s41390-020-1074-4 PMC8096612 32855506

[3] Fitzgibbons SC Ching Y Yu D. Mortality of necrotizing enterocolitis expressed by birth weight categories. J Pediatr Surg 2009;44:1072–75. 10.1016/j.jpedsurg.2009.02.013 19524719

[4] Zozaya C García González I Avila-alvarez A. Incidence, treatment, and outcome trends of necrotizing enterocolitis in preterm infants. Front Pediatr 2020;8:188. 10.3389/fped.2020.00188 PMC7237564 32478014

[5] Hu X Liang H Li F. Necrotizing enterocolitis: current understanding of prevention and management. Pediatr Surg Int 2024;40:32. 10.1007/s00383-023-05619-3 PMC10776729 38196049

[6] Kosloske AM. Pathogenesis and prevention of necrotizing enterocolitis: a hypothesis based on personal observation and review of the literature. Pediatrics 1984;74:1086–92. 6390330

[7] Moss RL Dimmitt RA Barnhart DC. Laparotomy versus peritoneal drainage for necrotizing enterocolitis and perforation. N Engl J Med 2006;354:2225–34. 10.1056/NEJMoa054605 16723614

[8] Petty JK Ziegler MM. Operative strategies for necrotizing enterocolitis. Semin Pediatr Surg 2005;14:191–98. 10.1053/j.sempedsurg.2005.05.009 16084407

[9] Struijs MC Poley MJ Meeussen CJ. Late versus early ostomy closure for necrotizing enterocolitis. J Pediatr Surg 2012;47:658–64. 10.1016/j.jpedsurg.2011.10.076 22498378

[10] SCARE Group, Kerwan A Al-Jabir A Mathew G. Revised Surgical CAse REport (SCARE) guideline: an update for the age of Artificial Intelligence. Prem J Sci 2025;10:100079.

[11] Pierro A Eaton S Ade-ajayi N. Peritoneal drainage as a surgical strategy for necrotizing enterocolitis: a systematic review. Arch Dis Child Fetal Neonatal Ed 2012;97:F456–F461.

[12] Zaczek Z Dąbrowska K Omidi M. Effect of thickening powder on gastrointestinal losses in patients with high-output jejunostomy syndrome. In Vivo 2022;36:884–89. 10.21873/invivo.12777 PMC8931876 35241546

[13] Vaughan WG Grosfeld JL West K. Avoidance of stomas and delayed anastomosis for bowel necrosis: the clip and drop-back technique. J Pediatr Surg 1996;31:542–45. 10.1016/s0022-3468(96)90492-3 8801309

[14] Beldowicz BC. The evolution of damage control in concept and practice. Clin Colon Rectal Surg 2018;31:30–35. 10.1055/s-0037-1602177 PMC5787400 29379405

[15] Neu J Walker WA. Necrotizing enterocolitis. N Engl J Med 2011;364:255–64. 10.1056/NEJMra1005408 PMC3628622 21247316

